# Supplementary material for: Interaction between Water and Wind as a Driver of Passive Dispersal in Mangroves
Source: PLoS One. 2015 Mar 26;10(3):e0121593. doi: 10.1371/journal.pone.0121593 (PMC4374773; doi:10.1371/journal.pone.0121593)

**Supporting Information for:**

**Interaction between Water and Wind as a Driver of Passive Dispersal in Mangroves**

Tom Van der Stocken^1,2,^*, Bram Vanschoenwinkel^1^, Dennis J.R. De Ryck^1,2^, Tjeerd J. Bouma^3^, Farid Dahdouh-Guebas^2,1^, Nico Koedam^1^

^1^ Laboratory of Plant Biology and Nature Management, Vrije Universiteit Brussel (VUB), Brussels, Belgium

^2^ Laboratory of Systems Ecology and Resource Management, Université Libre de Bruxelles (ULB), Brussels, Belgium

^3^ Department of Spatial Ecology, Royal Netherlands Institute for Sea Research (NIOZ), Yerseke, the Netherlands

* Corresponding author: tvdstock@gmail.com

**Table A.**

|  | **Mass (g)** | **Density (g l^-1^)** |
| --- | --- | --- |
| **SIZE A**  Length: 6 cm  Height: 4 cm | 8.12 | 159.20 |
|  | 9.52 | 186.68 |
|  | 21.91 | 429.55 |
|  | 45.71 | 897.40 |
| **SIZE B**  Length: 9 cm  Height: 5.5 cm | 20.63 | 160.19 |
|  | 23.42 | 182.97 |
|  | 54.70 | 420.78 |
|  | 115.76 | 883.66 |
| **SIZE C**  Length: 10 cm  Height: 7 cm | 42.12 | 160.67 |
|  | 47.39 | 180.42 |
|  | 112.27 | 428.92 |
|  | 240.66 | 905.79 |

**Table B.**

|  | SS | Df | MS | *F* | *P* |
| --- | --- | --- | --- | --- | --- |
| Intercept | 0.008978 | 1 | 0.008978 | 17.2985 | **0.000033** |
| Propagule density | 0.005142 | 1 | 0.005142 | 9.9077 | **0.001670** |
| Wind speed | 0.269978 | 1 | 0.269978 | 520.1850 | **<0.00001** |
| Water flow velocity | 0.090702 | 1 | 0.090702 | 174.7615 | **<0.00001** |
| Propagule density × Wind speed | 0.162847 | 1 | 0.162847 | 313.7676 | **<0.00001** |
| Propagule density × Water flow velocity | 0.009655 | 1 | 0.009655 | 18.6025 | **0.000017** |
| Wind speed × Water flow velocity | 0.001303 | 1 | 0.001303 | 2.5103 | 0.113259 |
| Propagule density × Wind speed × Water flow velocity | 0.003897 | 1 | 0.003897 | 7.5094 | **0.006192** |
| Error | 1.040084 | 2004 | 0.000519 |  |  |

**Table C.**

|  |  | **Wind speed** | | |
| --- | --- | --- | --- | --- |
|  |  | **L** | **M** | **H** |
| **Water flow velocity (m s^-1^)** | **0** | 38.57 ±14.37 | 29.82 ± 9.32 | 22.53 ± 7.52 |
|  | **0.15** | 11.25 ± 7.43 | 13.24 ± 4.97 | 17.29 ± 4.69 |
|  | **0.30** | 6.66 ± 3.84 | 5.77 ± 5.74 | 5.87 ± 5.41 |

**Figure A.**

**
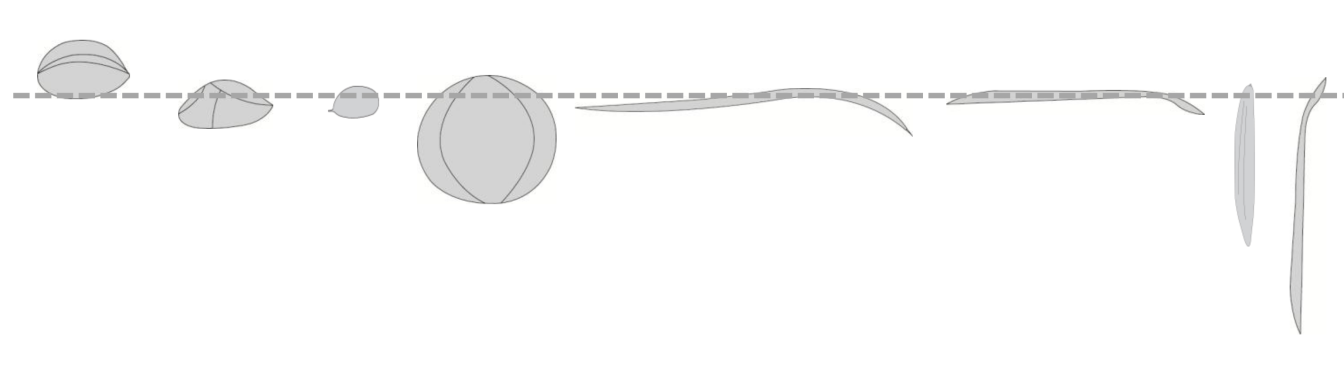
**

**Figure B.**





**Figure C.**


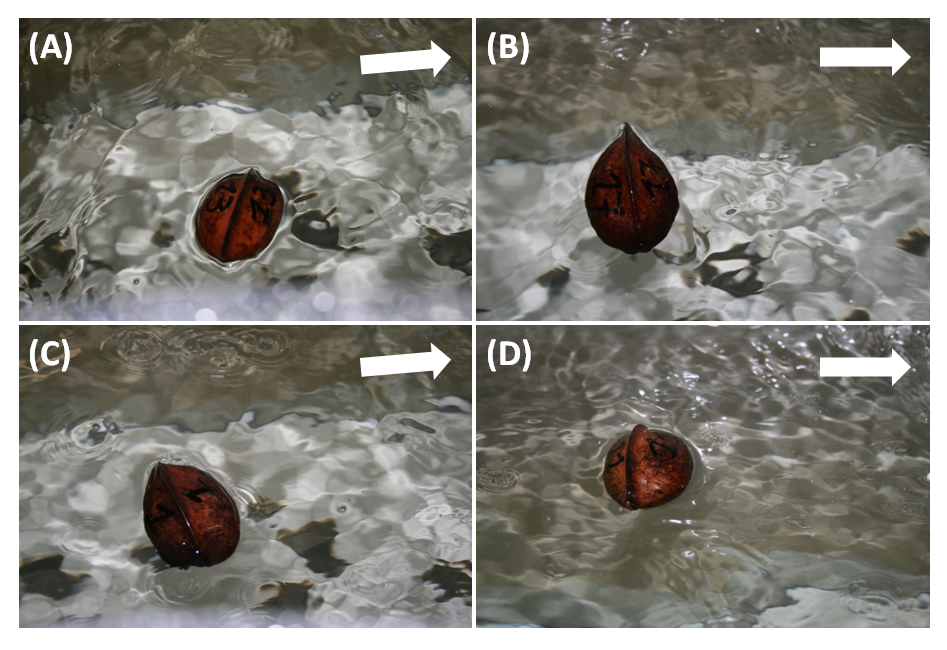

Supplement: S1 File — Table A: Characteristics of the egg-shaped mimics that were used to simulate Heritiera littoralis propagules without dorsal sail. Table B: Result of the general linear model for the effect of propagule density, wind speed, water flow velocity and the multiple interaction terms on dispersal velocity of mangrove propagules. Significant interactions (P < 0.05) are indicated in bold. Table C: Contribution of the dorsal sail of Heritiera littoralis in the total dispersal velocity (%). Densities of natural propagules were inserted in the regression line formulas for the mimicked sail-less H. littoralis propagules. As such, a proxy was obtained for their dispersal velocity in case they would not have a sail. Fig. A: Position of the mangrove propagule types used in his study relative to the water surface (dotted line). From left to right, represented propagules are from the following mangrove species: Heritiera littoralis, Xylocarpus granatum (seed), Avicennia marina, Xylocarpus granatum (fruit), Rhizophora mucronata, Ceriops tagal (horizontally floating), Bruguiera gymnorrhiza and Ceriops tagal (vertically floating). The scale of the propagules is not the same for all drawings. For the latter, the reader is referred to the propagule mean length data in Table 1 and values in Tomlinson [28]. Fig. B: Archived data on (A) wind speed and (B) wind direction, measured 3-hourly in Mombasa (http://www.wunderground.com). Data is presented over a one-year period, from 1 January 2013 to 1 January 2014. Dotted lines in (A) indicate wind speeds used in our flume study. Fig. C: Four different Heritiera littoralis propagules in the race-track flume. Water and wind currents are from left to right in all photographs (white arrow). All four propagules have a well-developed sail that is symmetrical to the transversal plane. During dispersal, and wind speeds being high enough, propagules typically have their sail oriented perpendicular to the wind force. All photographs taken by T. Van der Sto [file pone.0121593.s001.docx]
