# Supplementary material for: Correction: Interaction between Water and Wind as a Driver of Passive Dispersal in Mangroves
Source: PLoS One. 2015 Apr 22;10(4):e0127132. doi: 10.1371/journal.pone.0127132 (PMC4406683; doi:10.1371/journal.pone.0127132)
Supplement: S1 File — (PDF) [file pone.0127132.s001.pdf]

PLOS | ONE

RESEARCH ARTICLE

Interaction between Water and Wind as a Driver of Passive Dispersal in Mangroves

Tom Van der Stocken[1,2]*, Bram Vanschoenwinkel[1], Dennis J. R. De Ryck[1,2], Tjeerd J. Bouma[3], Farid Dahdouh-Guebas[2,1], Nico Koedam[1]

1 Laboratory of Plant Biology and Nature Management, Vrije Universiteit Brussel (VUB), Brussels, Belgium, **2** Laboratory of Systems Ecology and Resource Management, Université Libre de Bruxelles (ULB), Brussels, Belgium, **3** Department of Spatial Ecology, Royal Netherlands Institute for Sea Research (NIOZ), Yerseke, the Netherlands

* tvdstock@gmail.com

CrossMark
click for updates

OPEN ACCESS

Citation: Van der Stocken T, Vanschoenwinkel B, De Ryck DJR, Bouma TJ, Dahdouh-Guebas F, Koedam N (2015) Interaction between Water and Wind as a Driver of Passive Dispersal in Mangroves. PLoS ONE 10(3): e0121593. doi:10.1371/journal.pone.0121593

Academic Editor: Inés Álvarez, University of Vigo, SPAIN

Received: October 31, 2014

Accepted: February 11, 2015

Published: March 26, 2015

Copyright: © 2015 Van der Stocken et al. This is an open access article distributed under the terms of the Creative Commons Attribution License, which permits unrestricted use, distribution, and reproduction in any medium, provided the original author and source are credited.

Data Availability Statement: All relevant data are within the paper and its Supporting Information files.

Funding: This research was funded by the Flemish Interuniversity Council - University Development Cooperation (VLIR-UOS; http://www.vliruos.be/). TVdS has a VLIR PhD Scholarship (VLADOC). The funder had no role in study design, data collection and analysis, decision to publish, or preparation of the manuscript.

Competing Interests: The authors have declared that no competing interests exist.

Abstract

Although knowledge on dispersal patterns is essential for predicting long-term population dynamics, critical information on the modalities of passive dispersal and potential interactions between vectors is often missing. Here, we use mangrove propagules with a wide variety of morphologies to investigate the interaction between water and wind as a driver of passive dispersal. We imposed 16 combinations of wind and hydrodynamic conditions in a flume tank, using propagules of six important mangrove species (and genera), resulting in a set of dispersal morphologies that covers most variation present in mangrove propagules worldwide. Additionally, we discussed the broader implications of the outcome of this flume study on the potential of long distance dispersal for mangrove propagules in nature, applying a conceptual model to a natural mangrove system in Gazi Bay (Kenya). Overall, the effect of wind on dispersal depended on propagule density (g l^{-1}). The low-density *Heritiera littoralis* propagules were most affected by wind, while the high-density vertically floating propagules of *Ceriops tagal* and *Bruguiera gymnorrhiza* were least affected. *Avicennia marina*, and horizontally floating *Rhizophora mucronata* and *C*. *tagal* propagules behaved similarly. Morphological propagule traits, such as the dorsal sail of *H*. *littoralis*, explained another part of the interspecific differences. Within species, differences in dispersal velocities can be explained by differences in density and for *H*. *littoralis* also by variations in the shape of the dorsal sail. Our conceptual model illustrates that different propagule types have a different likelihood of reaching the open ocean depending on prevailing water and wind currents. Results suggest that in open water, propagule traits (density, morphology, and floating orientation) appear to determine the effect of water and wind currents on dispersal dynamics. This has important implications for inter- and intraspecific variation in dispersal patterns and the likelihood of reaching suitable habitat patches within a propagule's viable period.

Introduction

In many natural ecosystems, dispersal of organisms is mediated by a variety of external agents known as vectors such as wind, water and carrier animals. However, multiple vectors do not act independently [1,2,3]. For instance, wind may impact the flight patterns of birds transporting the seeds of wetland plants and the eggs of aquatic crustaceans. Similarly, local wind direction can constrain the transfer of genetic material through pollen by bumblebees. Insight in the multiple dispersal vectors involved in the dispersal process of a particular species is essential to realistically describe and predict dispersal trajectories [3]. In the case of oceanic dispersal, the course of dispersing propagules (i.e. dispersal units) is determined by the interaction of hydrodynamics and wind. However, this interaction has remained largely understudied, constraining the realism of existing dispersal models. Considering the wide variety of morphologically distinct propagules carried at the ocean surface [4], it is reasonable to assume that wind may differentially affect the dispersal patterns of these propagules. Such insight is highly relevant, especially in the context of habitat destruction and fragmentation which threaten biodiversity [5,6,7], since together with information on propagule viability it determines the probability of effective dispersal (*sensu* Nathan [8]). In this study, we use mangrove propagules with a wide variety of morphologies to test the effect of wind on hydrochorous dispersal. Mangroves appear along tropical and subtropical coasts where onshore and offshore winds could impact the fate of dispersing propagules, while the variety of morphologically distinct propagules allows us to study species-specific differential effects.

Given the seemingly infinite expanse of the world's oceans, transoceanic dispersal of mangrove tree species via specialized buoyant propagules can be considered a remarkable evolutionary achievement. Although most propagules disperse at a local scale, i.e. within the boundaries of the local habitat, a minority is exported to open water where they may contribute to long distance dispersal (LDD). A better understanding of dispersal distances and directions, i.e. dispersal patterns, is considered a priority given the increased fragmentation of natural mangrove habitats [9] and expected shifts of species ranges in response to global environmental change [10,11]. The latter requires populations to shift and settle a new population elsewhere or adapt to the new conditions. While dispersal within the local habitat drives local replenishment, LDD can be of disproportionate importance (with respect to numbers involved) by either mediating colonization of remote areas or by providing gene flow among distant populations, which can promote local adaptive potential. Additionally, rare LDD events across oceans can result in important biogeographic signals.

Dispersal distances of mangrove propagules have mostly been studied at local (hundreds of meters) and intermediate scales (several km) using marked propagules [12,13,14,15]. However, these release-recapture and genetic studies typically assume dispersal in a straight line from one location to another, and do not provide information on realized dispersal trajectories. At regional (10^3–10^5 m) and biogeographical (10^5–10^7 m) scales, quantifying dispersal poses methodological challenges [16,17]. Given the rare nature of LDD events, the time frame required for observation may be too long for most research programmes, while the dilution effect resulting from a low number of propagules spread over a vast expanse of water makes it practically unfeasible to intercept propagules during transport. Long-term echoes of rare dispersal events, however, can be detected in the population genetic structure [18,19,20]. Additionally, large-scale experiments such as the one performed by Steinke and Ward [21], in which 4500 drift cards were dropped from an aircraft into the sea, can help to demonstrate the feasibility of LDD. Geographic variation in allele frequencies, interception of propagules or recapture patterns of artificial propagules, however, typically do not generate information about the dispersal trajectories of individual propagules. In this context mechanistic models that integrate

information from ocean currents with intimate knowledge of mangrove ecology can play an important role. Although recent research shed new light on mangrove establishment requirements [22,23,24,25], the relative importance of many other traits that affect dispersal and mortality, remain obscure. Such knowledge, however, will not only be crucial to parameterize mechanistic models, it will also help to answer ecological questions such as to what extent the local species composition and diversity is controlled by dispersal limitation and the composition of the regional species pool (see Sutherland et al. [26]).

A largely neglected factor that could influence mangrove propagule dispersal dynamics is wind action [15]. A finite-volume advection-diffusion model developed by Di Nitto et al. [27] in a Sri Lankan lagoon complex suggested that wind action can affect dispersal trajectories. However, in this model, the authors applied a wind drag function uniformly on all species as a hydrodynamic component but species-specific differential effects were not considered [27]. Mangrove propagules strongly differ in propagule size, shape and density, which can affect the distribution of drag area inside and outside the water. Therefore, it is sensible to assume that the relative importance of wind versus water drag will differ strongly among species. In this study we build on our pilot study [15] in order to investigate general dispersal mechanisms across mangrove species. Additionally, the potential adaptive value of the dorsal sail of the mangrove species *Heritiera littoralis* in terms of promoting wind mediated hydrochorous dispersal has not yet been investigated. This notable morphological feature could facilitate or counteract hydrochorous dispersal depending on the relative direction of water and wind currents.

We used a racetrack flume adjusted with a wind generator to investigate variation in hydrochorous dispersal of mangrove propagules in response to different hydrodynamic and wind conditions. The experiment included propagules of six species and six genera, resulting in a set of morphologies that covers most variation present in mangrove propagules worldwide. In addition to the natural propagules, we used sail-less mimics of the characteristic sail-fitted propagules of *H. littoralis* to explore the potential adaptive origin of the dorsal sail in terms of its sensitivity to wind action. We hypothesized that (1) dispersal velocities are increasingly determined by wind speed and direction for propagules with decreasing density, because Archimedes' law dictates that they will have a higher proportional volume protruding from the water; (2) morphological traits that increase the wind drag outside the water, significantly enhance the effect of wind relative to the effect of water currents. The latter is expected to apply to propagules with a specific morphological feature, such as the *H. littoralis* propagules with a dorsal sail, as well as to propagules with a specific floating strategy, such as horizontally floating propagules compared to vertically floating ones. Finally, we discuss the broader implications of the outcome of this flume study on the potential for LDD, applying a conceptual model to a natural mangrove system.

Materials and Methods

Studied species

Species were selected to cover a wide range of morphological propagule types (Table 1; Fig. A in S1 File). The elongated (torpedo-shaped) propagules of *Ceriops tagal* (Perr.) C. B. Robinson and *Rhizophora mucronata* Lamk. (both Rhizophoraceae), strongly contrast with the ellipsoidal propagules of *Heritiera littoralis* Dryand. (Malvaceae). The raised dorsal sail [28], in combination with a very low density, ensures that *H. littoralis* propagules resemble small sailboats floating on the water surface. The cannonball-like fruits (a woody pericarp enclosing five to 20 seeds) of *Xylocarpus granatum* Koen. (Meliaceae) have much higher densities (983.64 ± 6.54 g l^{-1} compared to 726.33 ± 70.02 g l^{-1} for *H. littoralis*). As a result, the major part of their smooth

Table 1. Main propagule characteristics and overview of the dispersal velocities for the various hydrodynamic and wind treatments where wind and water acted in the same direction.

Species	*H. littoralis*	*X. granatum* seed	*A. marina*	*X. granatum* fruit	*R. mucronata*	*C. tagal*	*B. gymnorrhiza*	*C. tagal*
**Morphology**	Ellipsoidal	Angular/ Pyramidal	Ellipsoidal to flattened ovoid	Spherical ("cannonball")	Elongated	Elongated	Elongated	Elongated
**Floating orientation**	(-)	(-)	(-)	(-)	horizontal	horizontal	vertical	vertical
**n**	20	10	25	4	17	20	13	20
**Mean length (cm)**	(-)	(-)	(-)	(-)	36.45 ± 1.16	24.32 ± 2.14	16.02 ± 0.71	24.42 ± 3.23
**Mean mass (g)**	21.70 ± 0.93	58.00 ± 3.12	3.07 ± 0.10	943.51 ± 73.09	47.35 ± 2.42	7.28 ± 0.25	22.91 ± 1.57	7.08 ± 0.33
**Mean density (g l^{-1})**	726.33 ± 70.02	943.81 ± 17.79	968.10 ± 26.96	983.64 ± 6.54	1006.10 ± 5.76	1013.90 ± 8.04	1023.67 ± 5.23	1034.87 ± 7.20

For general information on the various propagule types, the reader is referred as well to data in Tomlinson [28].

doi:10.1371/journal.pone.0121593.t001

spherical body remains submerged. Besides the fruit, we also considered the irregular angular-shaped pyramidal seeds of *X. granatum*, since both the fruits and seeds of this species disperse in mangrove habitats. We complemented this selection with propagules of the important pioneer species *Avicennia marina* (Forssk.) Vierh. (Acanthaceae) and the elongated *Bruguiera gymnorrhiza* (L.) Lamk. (another member of the Rhizophoraceae). *Avicennia marina* propagules are ellipsoidal to flattened ovoid, small and light, floating at the water surface. They often carry their pericarp in the early stages of dispersal (personal observation). As for *C. tagal* and *R. mucronata*, *B. gymnorrhiza* propagules are viviparous (i.e. the embryo protrudes from the seed coat and the fruit, while attached to the parent tree; [28]) and typically elongated. *Rhizophora mucronata* has the largest propagules (36.45 ± 1.16 cm; n = 17), being much longer than *B. gymnorrhiza* propagules (16.02 ± 0.71 cm; n = 13), but having a comparable thickness. The propagules of *C. tagal* are the most slender, longer (24.37 ± 2.70 cm; n = 40) than *B. gymnorrhiza* propagules, and have a rough, warted and ribbed surface. It should be stressed here that differences in shape exist within the *C. tagal* and *R. mucronata* propagules, some being straight, while others can be bent near the plumule and the radicle. Whereas the floating orientation of *C. tagal* and *R. mucronata* propagules may vary between a horizontal and vertical position, *B. gymnorrhiza* propagules float vertically.

We used 20 horizontally and 20 vertically floating *C. tagal* propagules and 17 horizontally floating *R. mucronata* propagules. Vertically floating *R. mucronata* propagules were not considered since their length exceeded the water level in the flume, preventing vertical free flow. For *B. gymnorrhiza*, 13 vertically floating propagules were used. Furthermore, 25 *A. marina* (still carrying their pericarp) and 20 *H. littoralis* propagules were used. For *X. granatum*, we used four fruits and 10 individual seeds. All propagules were sampled in the mangrove forest of Gazi Bay, Kenya (39° 30' E, 4° 26' S). We measured the length and mass, and calculated the volume (using the water displacement method cf. Chave [29]) and density of all propagules. Propagules were checked for damage that could influence the buoyancy characteristics over the course of the experiments.

Propagule mimics

The potential adaptive origin of the dorsal sail in terms of its sensitivity to wind action, was tested using artificial propagules or mimics. These should be considered as *H. littoralis* propagules

without dorsal sail. The mimics consisted of plastic, egg-shaped dispersal items of various sizes, which were given different densities (per type, i.e. per size) by filling them with different loads of pebbles (see Table A in S1 File). Using a special silicone glue, the mimics were made waterproof to prevent their density from changing over the course of the experiments.

Flume study

A 17.5 m long and 0.6 m wide oval flume facility (Royal Netherlands Institute for Sea Research, NIOZ, Yerseke, The Netherlands) adjusted with an industrial ventilator was used to study the effect of wind on the dispersal velocity of hydrochorous mangrove propagules. This experimental set-up allowed for repetitions under controlled hydrodynamic and wind conditions. The flume was filled with seawater that was pumped directly from the sea next to the research institute. Water salinity and temperature were 35 ‰, and 9.6°C, respectively, yielding a water density of 1027.05 g l^{-1}. Water depth in the flume was kept constant at 0.36 m during the experiment. Using a conveyer belt, a unidirectional free flow current was generated. The smooth bottom (negligible bottom friction) of the flume ensures a steep water velocity gradient, simulating deeper water. An industrial ventilator was modified to allow for multiple wind speeds. To ensure wind speeds to be constant over the course of the experiments, the test section was covered with a plastic ceil and tested for leakages.

At each one-meter interval of the test section (5 m), wind speeds were measured with a velociCalc TSI anemometer (model 8384-M-GB) at three positions over the width of the flume (in the middle and at 0.15 m from both sides of the flume), i.e. 15 measurements in total. Water flow velocity measurements were taken with an Acoustic Doppler Velocimeter (ADV, Nortek AS, Oslo, Norway) placed on a 3D-positioning system.

We imposed 16 combinations of wind and hydrodynamic conditions: a unidirectional water flow (0.15 m s^{-1} and 0.30 m s^{-1}) without wind; a unidirectional water flow (0.15 m s^{-1} and 0.30 m s^{-1}) in combination with a low (ca. 2.5 m s^{-1}), medium (ca. 4.5 m s^{-1}) and high (ca. 6 m s^{-1}) wind speed in the same and opposite direction of the water flow; a low, medium and high wind speed without water flow. Water flow velocities and wind speeds were chosen to reflect conditions in a natural mangrove habitat, based on measurements by Kitheka, Ongwenyi and Mavuti [30] and archived weather data from Mombasa (Kenya) (see Fig. B in S1 File).

Propagules were released one by one at the start (0 m) of the test section and traveling times were recorded at each one-meter interval using a stopwatch. The first two meters of the test section were used for the propagules to reach an equilibrium dispersal velocity, and were not included in the calculations. Dispersal velocities were calculated, by dividing the time needed to travel over the last three meters of the test section (i.e. precautionarily excluding the first two meters to avoid possible instabilities which may be present near the ventilator). For the opposite wind treatments, calculations were made over the first three meters.

Conceptual model

A conceptual model for the potential of LDD for mangrove propagules in nature was constructed. We discuss the LDD potential of propagules released in Gazi Bay under different combinations of onshore vs. offshore water and wind currents (hypothetical scenarios). We do this both for propagules that are known to be affected by wind and for those that are relatively unaffected.

Data analysis

We conducted factorial Analysis of Variance (ANOVA) followed by pairwise Tukey post-hoc tests to investigate differences in dispersal velocity among and within species, for various

combinations of wind speed and water flow velocity. Additionally, a general linear model (GLM) was built with propagule density, wind speed and water flow velocity as continuous predictors for dispersal velocity. The GLM also contained the multiple interactions of these predictor variables. For investigating the effect of *H. littoralis'* dorsal sail in the wind-mediated hydrochorous dispersal process, dispersal velocity trend lines were calculated for the multiple mimics. These trend lines were then used to estimate dispersal velocities for densities of the natural *H. littoralis* propagules. Consequently, differences between the measured and estimated dispersal velocities served as a proxy for the contribution of the dorsal sail in the effect of wind. All statistical tests were performed in Statistica 8.0 (StatSoft, Inc.).

Results

Relevant propagule characteristics (morphology, floating orientation, mean length, mass and density) are summarized in Table 1 and Fig. A in S1 File. Mean propagule mass and densities ranged from 3.07 ± 0.10 g (*A. marina*) to 943.51 ± 73.09 g (*X. granatum* fruit), and from 726.33 ± 70.02 g l^{-1} (*H. littoralis*) to 1034.87 ± 7.20 g l^{-1} (vertically floating *C. tagal* propagules), respectively.

The average wind speed during the low (L), medium (M) and high (H) wind speed treatments was 2.77 ± 0.23 m s^{-1}, 4.53 ± 0.38 m s^{-1} and 6.03 ± 0.08 m s^{-1}, respectively. For the treatment where the wind direction was opposite to the water flow, wind speeds were slightly different since the ventilator had to be translocated and the construction with the ceil rebuilt: 2.68 ± 0.06 m s^{-1} (L), 4.55 ± 0.19 m s^{-1} (M) and 6.03 ± 0.05 m s^{-1} (H). Water flow velocities were 0 m s^{-1}, 0.16 ± 0.02 m s^{-1} and 0.31 ± 0.03 m s^{-1}.

The effect of wind on dispersal velocities was strongly different among propagule types in the treatment without water flow (factorial ANOVA, $P < 0.0001$, $F_{6.341} = 442.48$, adjusted $R^2 = 0.93$) as well as under the 0.15 m s^{-1} (factorial ANOVA, $P < 0.0001$, $F_{7.715} = 46.17$, adjusted $R^2 = 0.98$) and 0.30 m s^{-1} (factorial ANOVA, $P < 0.0001$, $F_{7.829} = 28.54$, adjusted $R^2 = 0.96$) water flow velocity treatment. *Heritiera littoralis* propagules responded stronger to imposed wind speeds than other propagule types (Fig. 1). Interestingly, in the treatment with the high water flow velocity and low wind speed in the same direction, *H. littoralis* propagules were the only propagule type of which the dispersal velocity was strongly affected by wind action. They showed higher dispersal velocities than all other propagule morphotypes (One-way ANOVA, $P < 0.0001$, $F_{1.126} = 317.80$, adjusted $R^2 = 0.714$). The dispersal velocity of the vertically floating *C. tagal* and *B. gymnorrhiza* propagules were equally affected by wind action in all water flow velocity treatments (0 m s^{-1}: One-way ANOVA, $P = 0.0706$, $F_{1.61} = 3.39$; 0.15 m s^{-1}: One-way ANOVA, $P = 0.8847$, $F_{1.186} = 0.02$; 0.30 m s^{-1}: One-way ANOVA, $P = 0.4006$, $F_{213} = 0.71$). In all wind speed treatments these propagule types were less affected by wind than the other propagule types (Fig. 1). Wind equally affected the dispersal velocities of the horizontally floating propagules of *C. tagal* and *R. mucronata* under all water flow velocity conditions (0 m s^{-1}: One-way ANOVA, $P = 0.1830$, $F_{1.109} = 1.80$; 0.15 m s^{-1}: One-way ANOVA, $P = 0.4734$, $F_{1.219} = 0.51$; 0.30 m s^{-1}: One-way ANOVA, $P = 0.2032$, $F_{1.255} = 1.63$). The effect of wind on the dispersal velocity of *A. marina* propagules is similar to that on the dispersal velocity of the horizontally floating propagules of *C. tagal* and *R. mucronata* (Fig. 1), while the fruit of *X. granatum* generally shows dispersal velocities that are higher than that of the vertically floating *C. tagal* and *B. gymnorrhiza* propagules, but lower than that of all the other propagule types. The *X. granatum* seeds experience less influence from wind than *H. littoralis*, but slightly more than *A. marina* and the horizontally floating *C. tagal* and *R. mucronata* propagules.

The general linear model ($F = 5494.98$, $P < 0.001$, adjusted $R^2 = 0.95$) showed both significant main effects of density, water flow velocity and wind speed on dispersal velocity as well as

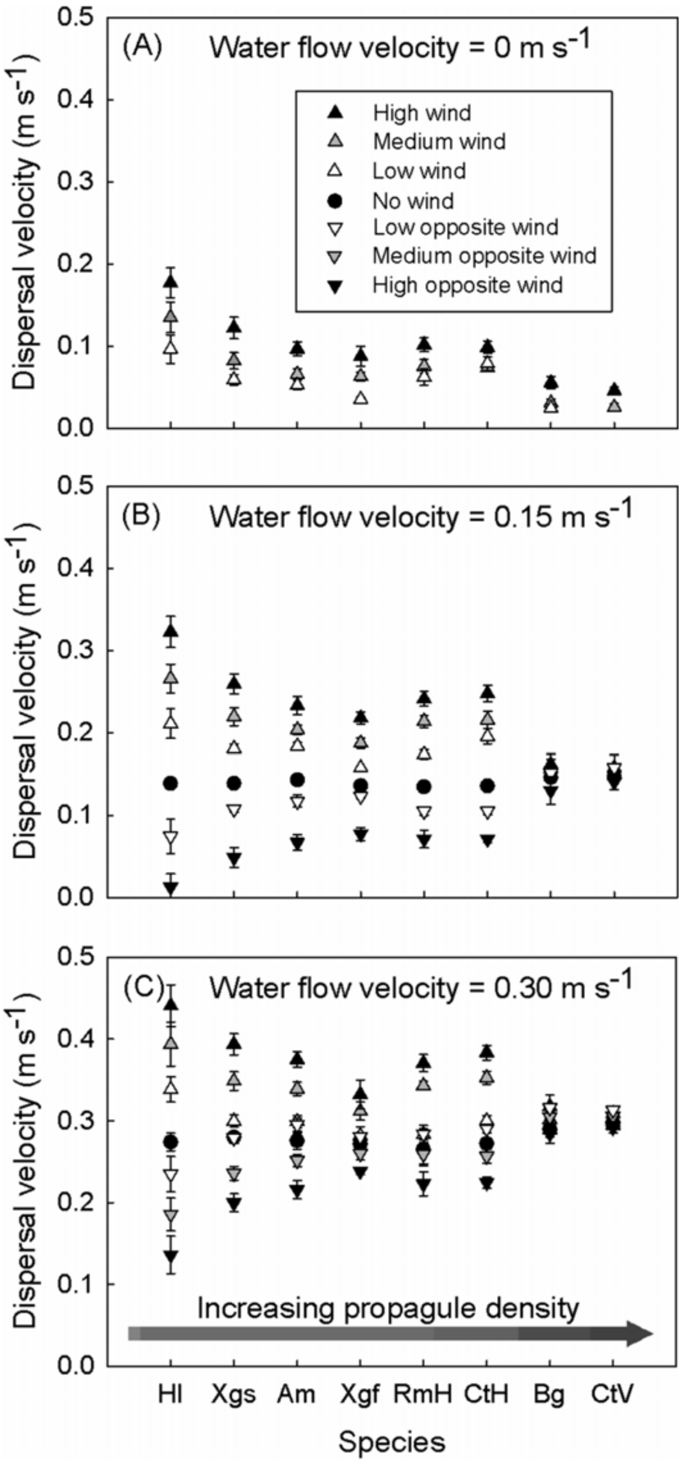

Fig 1. Dispersal velocities (y-axis) of the propagules used in this study (x-axis), under various wind conditions for three different water flow velocities: (A) 0 m s^{-1}, (B) 0.15 m s^{-1} and (C) 0.30 m s^{-1}. Dispersal units on the x-axis are ranked from lowest (left) to highest (right) density, as indicated by the arrow. Hl: *Heritiera littoralis*; XgS: *Xylocarpus granatum* seed; Am: *Avicennia marina*; Xgf: *X*. *granatum* fruit; RmH: horizontally floating *Rhizophora mucronata*; CtH: horizontally floating *Ceriops tagal*; Bg: *Bruguiera gymnorrhiza*; CtV: vertically floating *C*. *tagal*.

doi:10.1371/journal.pone.0121593.g001

Table 2. Results of the general linear model for the effect of propagule density, wind speed, water flow velocity and the multiple interaction terms on dispersal velocity.

	Dispersal velocity Parameter	Dispersal velocity Std. Err.	Dispersal velocity t	Dispersal velocity *P*
Intercept	**0.052799**	**0.012695**	**4.1591**	**0.000033**
Propagule density	**-0.000042**	**0.000013**	**-3.1476**	**0.001670**
Wind speed	**0.063756**	**0.002795**	**22.8076**	<**0.00001**
Water flow velocity	**0.679794**	**0.051423**	**13.2197**	<**0.00001**
Propagule density × Wind speed	**-0.000052**	**0.000003**	**-17.7135**	<**0.00001**
Propagule density × Water flow velocity	**0.000231**	**0.000054**	**4.3131**	**0.000017**
Wind speed × Water flow velocity	0.018042	0.011387	1.5844	0.113259
Propagule density × Wind speed × Water flow velocity	**-0.000032**	**0.000012**	**-2.7403**	**0.006192**
Error	**1.04**			

Significant interactions ($P < 0.05$) are indicated in bold.

doi:10.1371/journal.pone.0121593.t002

interactive effects (Table 2; Table B in S1 File). The model included two significant two-way interactions as well as a significant three-way interaction. Overall, water flow velocity and (positive, in line with water flow velocity) wind speed promoted dispersal velocity, while negative wind speeds decreased dispersal velocity. Particularly lower density propagules were most sensitive to the wind treatments. Depending on the direction of the water flow vs. air flow, propagules exhibited acceleration (same direction) or deceleration (opposite direction) of their dispersal velocity (Figs. 1 and 2). Significant interaction terms in the model support the interpretation of the effect of water flow velocity and wind speed being dependent on propagule density (Table 2; Table B in S1 File).

Overall, *H. littoralis* propagules with a sail responded stronger to wind than the egg-shaped mimics with a similar density but without such structures (Fig. 3). An indication of the contribution of the dorsal sail in the total dispersal velocity is summarized in Table C in S1 File.

Discussion

Predicting dispersal trajectories requires substantial knowledge on the multiple dispersal vectors involved [3,17]. Although the idea that wind action may modulate hydrochorous dispersal is widely held [27,31,32,33,34], the concept has rarely been tested for mangrove propagules (but see Van der Stocken et al. [15]). The present study considers a wide range of natural wind and hydrodynamic conditions and includes propagule morphotypes that cover most variation present in mangrove propagules worldwide as well as mimics, allowing for a generic across-species understanding of which factors control dispersal.

The role of propagule density

In the absence of wind, all propagules dispersed at velocities close or equal to the water flow velocity (Fig. 1; the treatment with a 0 m s^{-1} water flow and "No wind" was not considered since no dispersal vectors act on the propagules in that case). Only the horizontally floating *C. tagal* and *R. mucronata* propagules and the fruits of *X. granatum* seemed to disperse slightly slower. This may be due to a lower drag force at the propagule surface-water contact because of their smooth surface and streamlined shape. Adding wind to the experimental set-up, however, resulted in important changes in the relative dispersal velocities of different propagule types (Fig. 1). In all treatments the propagules of *H. littoralis* were most influenced by wind, while

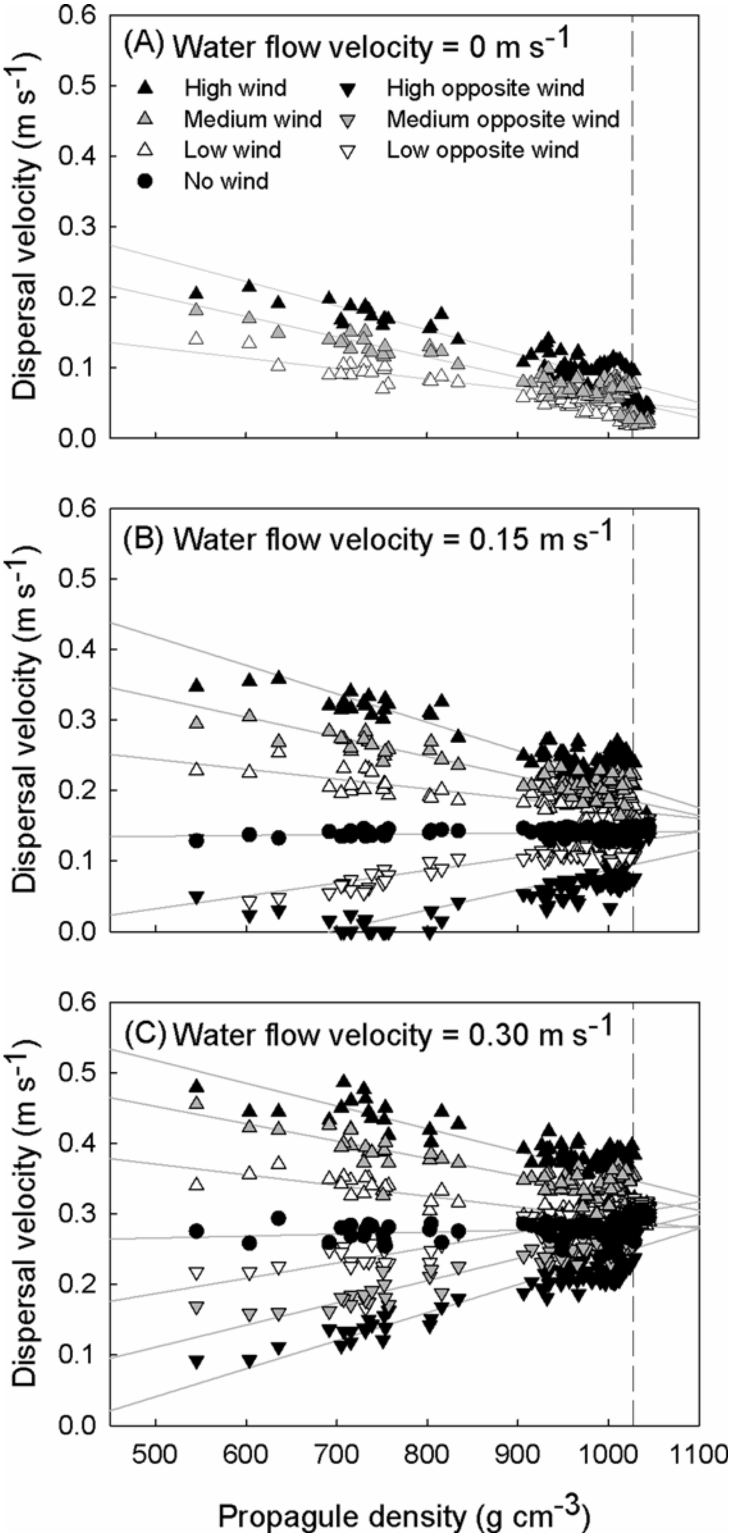

Fig 2. Dispersal velocities (y-axis) for all propagules used in this study, as a function of propagule density (x-axis), under various wind conditions for three different water flow velocities: (A) 0 m s^{-1}, (B) 0.15 m s^{-1} and (C) 0.30 m s^{-1}. Regression lines are plotted in light grey. The vertical dashed line indicates the water density (1027.05 g l^{-1}).

doi:10.1371/journal.pone.0121593.g002

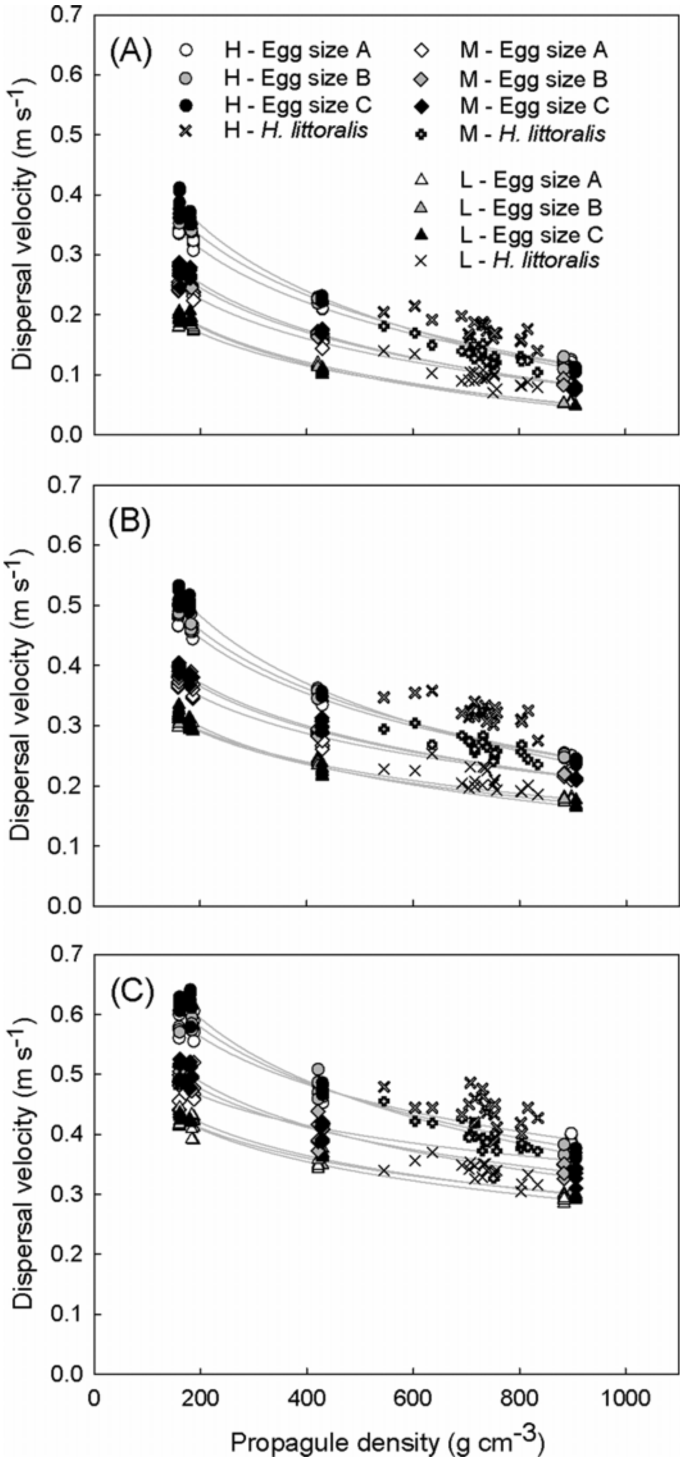

Fig 3. Dispersal velocities of sail-less egg-shaped propagule mimics and natural *Heritiera littoralis* propagules under various wind conditions for three different water flow velocities: (A) 0 m s^{-1}, (B) 0.15 m s^{-1} and (C) 0.30 m s^{-1}. Mimics of three different sizes with three different densities for each size were used (see Table A in S1 File). These mimics were used to simulate *H. littoralis* propagules without apical sail. Multiple wind speeds were imposed (L: low = 2.77 ± 0.23 m s^{-1}; M: medium = 4.53 ± 0.38 m s^{-1}; H: high = 6.03 ± 0.08 m s^{-1}) to the propagules. Trend lines were added for the mimics (light grey) for comparison with the natural propagules.

doi:10.1371/journal.pone.0121593.g003

the dispersal velocity of the vertically floating *B. gymnorrhiza* and *C. tagal* propagules were least influenced. Differences in propagule density appear to be a crucial determinant for the effect of wind on dispersal trajectories (Table 2; Fig. 2). This can be explained by Archimedes' law, since lower density propagules (*H. littoralis* propagules) will have a higher proportion of their volume protruding above the water surface than higher density propagules (cf. vertically floating *C. tagal* and *B. gymnorrhiza* propagules). This proportion determines the area on which ambient wind forces can exert a drag force. Propagules with a density close to that of the water such as the vertically floating *C. tagal* and *B. gymnorrhiza* propagules do not protrude from the water and hence are largely unaffected by direct wind action (Fig. 2). Similar effects of propagule density are confirmed by the GLM. Significant interaction terms show that the effects of wind and water speed are confounded by propagule density. Unlike seeds in other systems [35,36] mangrove propagules do not differ in terms of water saturation (dry or waterlogged). Hence, this cannot influence their density and their buoyancy behaviour. The floating orientation of *C. tagal* and *R. mucronata*, however, can change with time [37] resulting in a different susceptibility to wind. Whether these species can change their floating capacity after drying or after sinking and re-exposure is currently unknown. Long-term flotation experiments could shed new light on this process. Additionally, estimates of the overall fecundity and knowledge on the proportions of vertically and horizontally floating propagules at the moment following abscission would be beneficial for the quality of dispersal models.

Average water temperature and salinity values for coastal tropical water are different from those of the water used in our flume study. Additionally, water properties may change considerably over the course of a propagule's dispersal trajectory. Taking an average water temperature of 20°C and a salinity of 36 ‰ for tropical coastal water, the water density would be 1025.55 g l^{-1} instead of 1027.05 g l^{-1} in our flume study. We think that the effect on the emerged propagule portion would be minor, and the impact on the effect of wind negligible. For propagules with a density close to that of the water, changes in water temperature and salinity may affect the threshold between sinking or floating. However, for the purpose of this study, we deliberately focused on propagules that float. Sunken propagules under tropical water conditions would not have been taken into account.

Impact of propagule morphology

Pronounced variation in dispersal velocities were found among the twenty *H. littoralis* propagules studied, depending on the wind treatment. While variations in density, which range from 545.12 to 834.22 g l^{-1}, may explain part of this variation, the presence of a dorsal sail increased the effect of wind (Fig. 3; Table C in S1 File). *Heritiera littoralis* propagules with a well developed sail that is symmetrical to the transversal plane, typically float with their sail perpendicular to the wind (see Fig. C in S1 File), while propagules with an asymmetrical sail show stable orientations at sub-orthogonal (i.e. $< 90°$) attack angles. Propagules with an underdeveloped sail are less affected by wind forces. Considering the presence of similar sail-like structures in the seafaring colonial cnidarian animals *Physalia physalia* (L.) [38] and *Velella velella* (L.) [39], it is sensible to assume that the sail of *H. littoralis* consists of an adaptive trait to make use of wind forces and compete with other mangrove species which lack such adaptations. This dispersal process with a strong sailing component would be called 'pleustochory' rather than mere hydrochory.

Morphological traits were not studied in the other mangrove species. However, the small standard deviations make it reasonable to assume that morphological trait variation within these species will be of minor importance. Conversely, some of our findings suggest that differential effects of wind among species could be explained by morphological features. For

example, while *X. granatum* fruits dispersed faster than the vertically floating *C. tagal* and *R. mucronata* propagules, they moved slower than the other propagule types. Since balance of the propagules with ambient dispersal vectors was ensured, the lower dispersal velocity of these fruits may result from a lower drag force at the propagule surface-water contact, but may at least partly result from the smooth spherical shape of the propagules which results in reduced mechanical friction. Similarly, the angular shaped *X. granatum* seeds have a rougher above-water surface which, via higher mechanical friction, may explain the stronger effects of wind on their dispersal velocity than the other dispersal units (except *H. littoralis*).

Implications on dispersal patterns

Habitat destruction and fragmentation, as well as climate change alter the spatial configuration of suitable and unsuitable habitats [40]. Therefore, knowledge on dispersal distances and direction, LDD in particular, is essential as it allows to assess and predict the probability of propagules to reach and colonize remote habitat fragments [17,41]. Evidence for the ability of species to disperse over long distances via ocean currents dates back to the flotation experiments of Darwin [42], but challenges related to direct observations and the stochasticity associated with

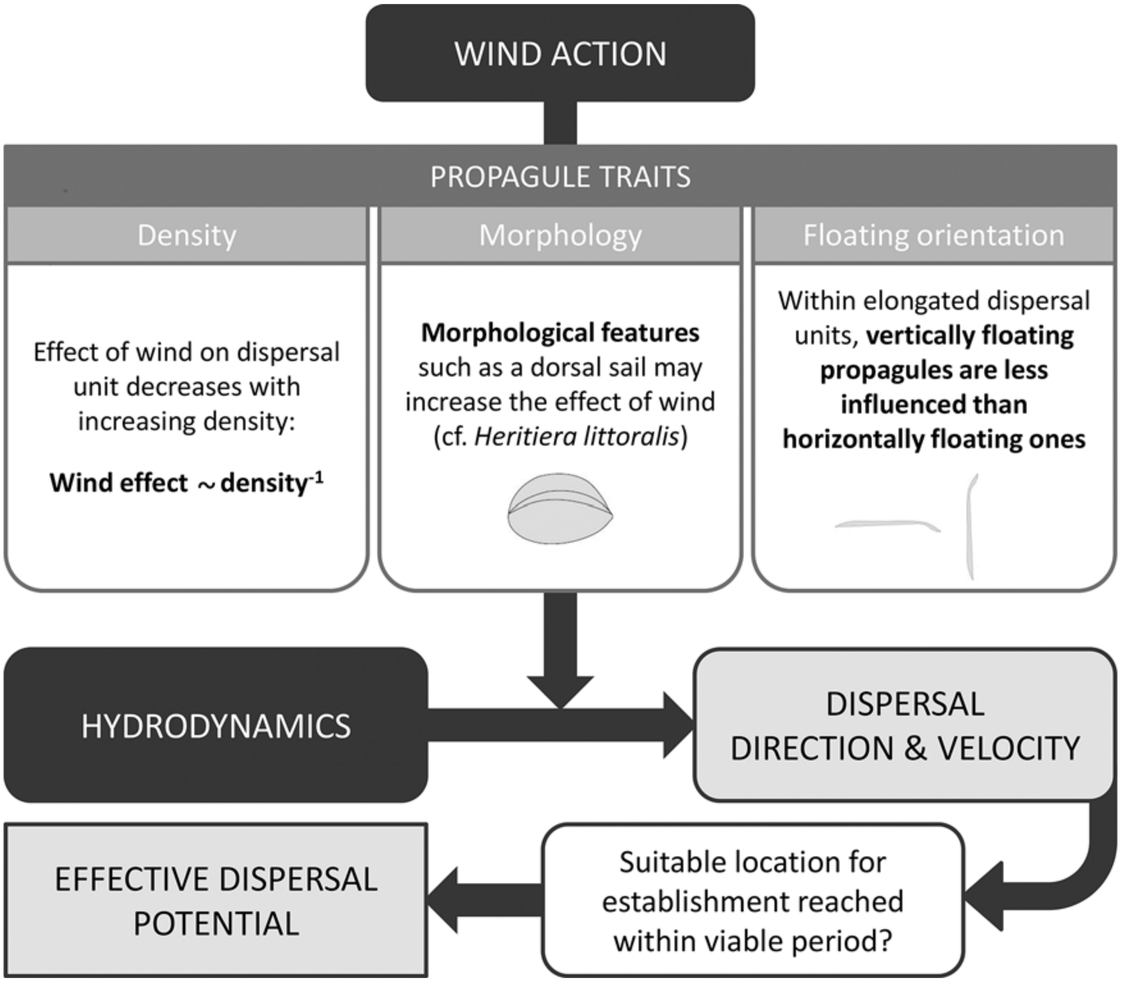

Fig 4. Diagram indicating how hydrodynamic and wind forces determine the dispersal direction and velocity of propagules, and in combination with the viable period of these propagules determine effective dispersal potential. The effect of wind depends on multiple propagule traits.

doi:10.1371/journal.pone.0121593.g004

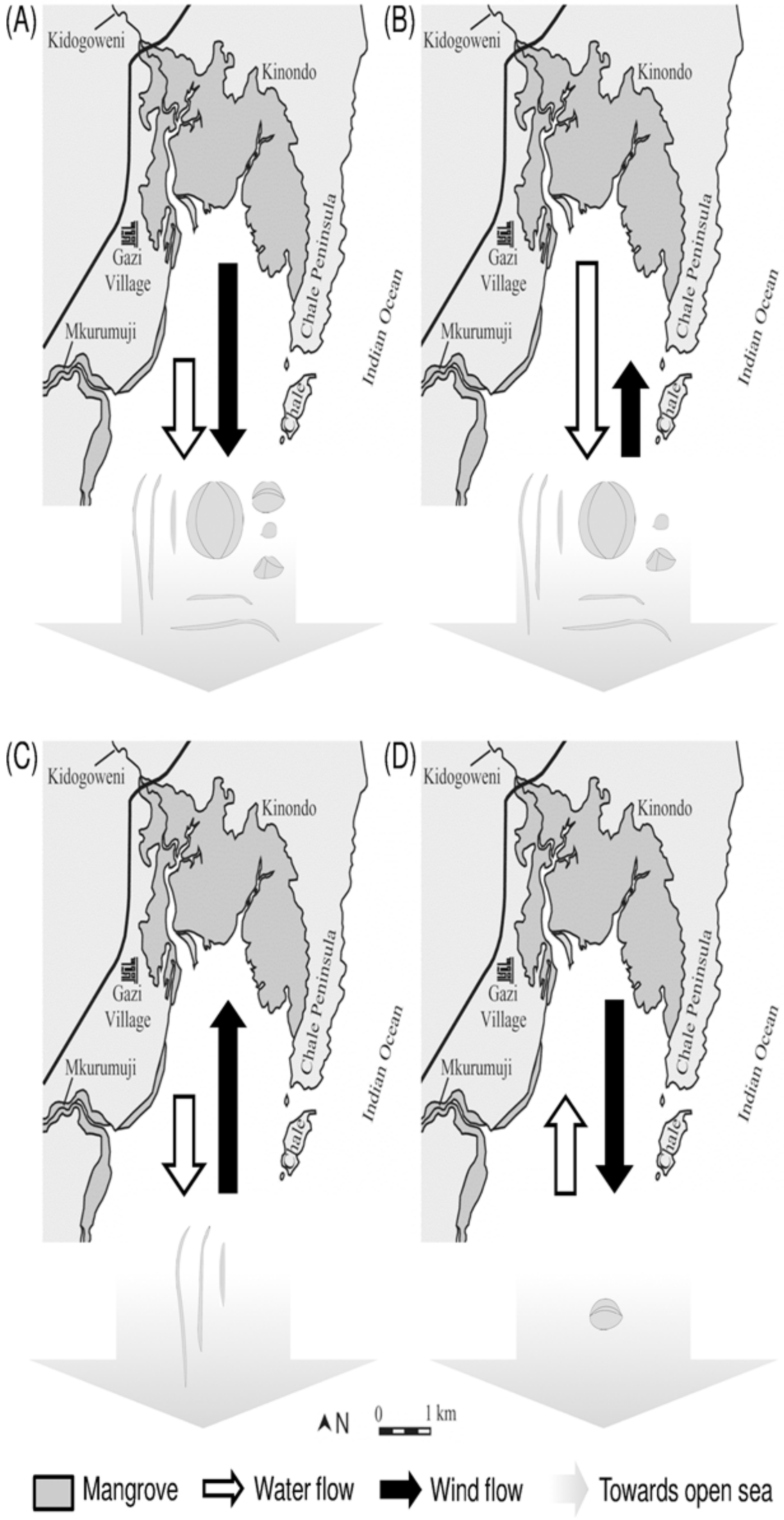

Fig 5. Conceptual representation of how the interplay between water and wind currents may influence the potential for long distance dispersal of mangrove propagules in Gazi Bay (Kenya). When both dispersal vectors are parallel and in the same direction (A), towards the open ocean, all propagules could leave the local system. In case of strong offshore ocean currents and onshore winds, all propagules with the exception of *H*. *littoralis*, will be able to escape (B). When offshore water currents are weak, strong onshore

winds may constitute an important barrier for propagules that float at or on the water surface, hindering them from reaching the ocean (C). Deeply submerged propagules are less affected. When ocean currents are onshore and offshore winds are strong, only *H*. *littoralis* propagules will be able to embark on LDD (D). The map of Gazi Bay is modified after Dahdouh-Guebas et al. [43].

doi:10.1371/journal.pone.0121593.g005

LDD hamper the quantification and prediction of such events [8], and constrain the realism of dispersal models. As stressed by Nathan [8] the best way to tackle this problem is to focus on the mechanisms involved. For passive dispersers at the ocean surface, the most straightforward factor to consider when predicting dispersal patterns is hydrodynamics. However, in this study we clearly demonstrate that in such systems, wind can modulate dispersal trajectories depending on propagule density and specific morphological features. Besides average dispersal patterns and the probability of propagules to leave the local habitat and embark on LDD, it determines the likelihood of propagules to reach a suitable location within their viable period, i.e. the potential of effective dispersal (Fig. 4). The implications of our findings for the potential of LDD are schematically illustrated for a mangrove system in Gazi Bay, Kenya (Fig. 5). When outgoing water flow coincides with (strong) northerly winds (Fig. 5A), or when the outgoing water currents are strong compared to southerly winds (Fig. 5B), all propagule types could reach the open ocean. However, *H*. *littoralis* propagules would disperse slowly or be prevented from leaving the local system as its dorsal sail allows prevailing wind forces to counteract the effect of hydrodynamics. When outgoing water flow is weak and strong winds act from the south, the elongated vertically floating propagules would be the only propagule types able to reach the Indian ocean and embark on LDD (Fig. 5C). A low density and specific morphological features may render some propagules more efficient at reaching the Indian Ocean when strong northerly winds overrule the effect of onshore water flow (Fig. 5D). For the mangrove system depicted in Fig. 5, the average daily wind direction from 1 January 2013 to 1 January 2014 is shown in Fig. B in S1 File. While wind predominantly comes from the northeast from early December to late February, wind comes from the south during most of the year. This strongly limits the opportunity for most propagule types to leave this mangrove system. However, they may do so during windows of lower wind speeds.

Conclusion

In conclusion, our study demonstrates that propagule density and morphology exert strong control on the way wind influences the dynamics of hydrochorous propagules floating at the surface of oceans and seas. For realistically predicting dispersal patterns, hydrochorous dispersal models should include species-specific differential effects of wind based on propagule traits. Additionally, information on both the floating and viable period of propagules is needed, since these factors represent temporal constraints to the potential of effective dispersal. Viable propagules that sink before reaching a suitable site, or propagules that reach a suitable location but are no longer viable, do not contribute to effective dispersal. Eventually, the present species-specific results on propagule dispersal properties will have consequences for long-term population dynamics, biogeographical ranges, connectivity patterns, and phenomena such as the failure of species to fully exploit their potential ranges based on niche models [44].

Supporting Information

S1 File. Combined file of supporting information. Table A: Characteristics of the egg-shaped mimics that were used to simulate *Heritiera littoralis* propagules without dorsal sail. **Table B**: Result of the general linear model for the effect of propagule density, wind speed, water flow velocity and the multiple interaction terms on dispersal velocity of mangrove propagules.

Significant interactions ($P < 0.05$) are indicated in bold. **Table C**: Contribution of the dorsal sail of *Heritiera littoralis* in the total dispersal velocity (%). Densities of natural propagules were inserted in the regression line formulas for the mimicked sail-less *H. littoralis* propagules. As such, a proxy was obtained for their dispersal velocity in case they would not have a sail. **Fig. A**: Position of the mangrove propagule types used in his study relative to the water surface (dotted line). From left to right, represented propagules are from the following mangrove species: *Heritiera littoralis*, *Xylocarpus granatum* (seed), *Avicennia marina*, *Xylocarpus granatum* (fruit), *Rhizophora mucronata*, *Ceriops tagal* (horizontally floating), *Bruguiera gymnorrhiza* and *Ceriops tagal* (vertically floating). The scale of the propagules is not the same for all drawings. For the latter, the reader is referred to the propagule mean length data in Table 1 and values in Tomlinson [28]. **Fig. B**: Archived data on (A) wind speed and (B) wind direction, measured 3-hourly in Mombasa (http://www.wunderground.com). Data is presented over a one-year period, from 1 January 2013 to 1 January 2014. Dotted lines in (A) indicate wind speeds used in our flume study. **Fig. C**: Four different *Heritiera littoralis* propagules in the race-track flume. Water and wind currents are from left to right in all photographs (white arrow). All four propagules have a well-developed sail that is symmetrical to the transversal plane. During dispersal, and wind speeds being high enough, propagules typically have their sail oriented perpendicular to the wind force. All photographs taken by T. Van der Stocken. (DOCX)

Acknowledgments

Authors are very grateful to the technical staff of the Royal Netherlands Institute for Sea Research (NIOZ) for their technical assistance in the flume experiments. Special thanks are due to Mr. Hamisi Kirauni for collecting propagules in the mangrove forest of Gazi Bay. We thank Dr. J. G. Kairo of the Kenya Marine and Fisheries Research Institute (KMFRI) for his logistic support during our 2012 fieldwork mission.

Author Contributions

Conceived and designed the experiments: TVdS DJRDR TJB. Performed the experiments: TVdS DJRDR. Analyzed the data: TVdS. Contributed reagents/materials/analysis tools: TVdS TJB. Wrote the paper: TVdS DJRDR BV NK. Supervised the research: NK FDG TJB. Provided substantial revisions: TVdS BV NK TJB DJRDR FDG.

References

1. van der Pijl L. Principal of dispersal in higher plants. Springer-Verlag, Berlin; 1982.
2. Ozinga WA, Bekker RM, Schaminée JHJ, van Groenendael JM. Dispersal potential in plant communities depends on environmental conditions. Journal of Ecology. 2004; 92: 767–777.
3. Nathan R. Total dispersal kernels and the evaluation of diversity and similarity in complex dispersal systems. In: Dennis AJ, Schupp EW, Green RJ, Westcott DA, editors. Seed dispersal: theory and its application in a changing world. Oxfordshire: CAB International Publishing; 2007. pp. 252–276.
4. Gunn CR, Dennis JV. World guide to tropical drift seeds and fruits. Malabar, Florida: Krieger Publishing Company; 1976.
5. Tilman D, May RM, Lehman CL, Nowak MA. Habitat destruction and the extinction debt. Nature. 1994; 371: 65–66.
6. Fahrig L. Effects of habitat fragmentation on biodiversity. Annual Review of Ecology and Systematics. 2003; 34: 487–515.
7. Ewers RM, Didham RK. Confounding factors in the detection of species responses to habitat fragmentation. Biological Reviews. 2006; 81: 117–142. PMID: 16318651
8. Nathan R. Long distance dispersal of plants. Science. 2006; 313: 786–788. PMID: 16902126

9. Duke NC, Meynecke JO, Dittmann S, Ellisson AM, Anger K, Berger U, et al. A World Without Mangroves? Science. 2007; 317: 41–43. PMID: 17615322

10. Valiela I, Bowen JL, York JK. Mangrove forests: One of the world's threatened major tropical environments. Bioscience. 2001; 51: 807–815.

11. Gilman EL, Ellison J, Duke NC, Field C. Threats to mangroves from climate change and adaptation options: A review. Aquatic Botany. 2008; 89: 237–250.

12. Yamashiro M. Ecological study on *Kandelia candel* (L) Druce, with special reference to the structure and falling of the seedlings. Hikobia. 1961; 2: 209–214.

13. Chan HT, Husin N. Propagule dispersal, establishment and survival of *Rhizophora mucronata*. The Malaysian Forester. 1985; 48: 324–329.

14. Breitfuss MJ, Connolly RM, Dale PER. Mangrove distribution and mosquito control: transport of *Avicennia marina* propagules by mosquito-control runnels a in southeast Queensland saltmarshes. Estuarine Coastal and Shelf Science. 2003; 56: 573–579.

15. Van der Stocken T, De Ryck DJR, Balke T, Bouma TJ, Dahdouh-Guebas F, Koedam N. The role of wind in hydrochorous mangrove propagule dispersal. Biogeosciences. 2013; 10: 895–925.

16. Nathan R. The challenges of studying dispersal. Trends in Ecology & Evolution. 2001; 16: 481–483.

17. Nathan R, Schurr FM, Spiegel O, Steinitz O, Trakhtenbrot A, Tsoar A. Mechanisms of long-distance seed dispersal. Trends in Ecology and Evolution. 2008; 23: 638–647. doi: 10.1016/j.tree.2008.08.003 PMID: 18823680

18. Dodd RS, Afzal-Rafii Z, Kashani N, Budrick J. Land barriers and open oceans: effects on gene diversity and population structure in *Avicennia germinans* L. (Avicenniaceae). Molecular Ecology. 2002; 11: 1327–1338. PMID: 12144655

19. Nettel A, Dodd RS. Drifting propagules and receding swamps; genetic footprints of long—distance dispersal and Quaternary extinction along tropical coasts. Evolution. 2007; 61: 958–971. PMID: 17439624

20. Marris E. Tree hitched a ride to island. Nature. 2014; 510: 320–321. doi: 10.1038/510320a PMID: 24943937

21. Steinke TD, Ward CJ. Use of plastic drift cards as indicators of possible dispersal of propagules of the mangrove *Avicennia marina* by ocean currents. African Journal of Marine Science. 2003; 25: 169–176.

22. Balke T, Bouma TJ, Horstman EM, Webb EL, Erftemeijer PLA, Herman PMJ. Windows of opportunity: thresholds to mangrove seedling establishment on tidal flats. Marine Ecology Progress Series. 2011; 440: 1–9.

23. Balke T, Bouma TJ, Herman PMJ, Horstman EM, Sudtongkong C, Webb EL. Cross-shore gradients of physical disturbance in mangroves: implications for seedling establishment. Biogeosciences. 2013; 10: 5411–5419.

24. Balke T, Webb EL, van den Elzen E, Galli D, Herman PMJ, Bouma TJ. Seedling establishment in a dynamic sedimentary environment: a conceptual framework using mangroves. Journal of Applied Ecology. 2013; 50: 740–747. PMID: 23894211

25. Balke T, Herman PMJ, Bouma TJ. Critical transitions in disturbance-driven ecosystems: identifying Windows of Opportunity for recovery. Journal of Ecology. 2014; 102: 700–708.

26. Sutherland WJ, Freckleton RP, Godfray HCJ, Beissinger SR, Benton T, Cameron DD. Identification of 100 fundamental ecological questions. Journal of Ecology. 2013; 101: 58–67.

27. Di Nitto D, Erftemeijer PLA, van Beek JKL, Dahdouh-Guebas F, Higazi L, Quisthoudt K. Modelling drivers of mangrove propagule dispersal and restoration of abandoned shrimp farms. Biogeosciences. 2013; 10: 5095–5113.

28. Tomlinson PB. The Botany of Mangroves. Cambridge: Cambridge University Press; 1986.

29. Chave J. Measuring Wood Density for Tropical Forest Trees: A Field Manual for CTFS Sites. Toulouse, France: Université Paul Sabatier; 2005.

30. Kitheka JU, Ongwenyi GS, Mavuti KM. Fluxes and exchange of suspended sediment in tidal inlets draining a degraded mangrove forest in Kenya. Estuarine Coastal and Shelf Science. 2003; 56: 655–667.

31. Clarke PJ. Dispersal of grey mangrove (*Avicennia marina*) propagules in southeastern Australia. Aquatic Botany. 1993; 45: 195–204.

32. de Lange WP, de Lange PJ. An appraisal of factors controlling the latitudinal distribution of mangrove (*Avicennia marina* var. *resinifera*) in New Zealand. Journal of Coastal Research. 1994; 10: 539–548.

33. Stieglitz T, Ridd PV. Trapping of mangrove propagules due to density-driven secondary circulation in the Normanby River estuary, NE Australia. Marine Ecology Progress Series. 2001; 211: 131–142.

34. Sarneel JM, Beltman B, Buijze A, Groen R, Soons MB. The role of wind in the dispersal of floating seeds in slow-flowing or stagnant water bodied. Journal of Vegetation Science. 2014; 25: 262–274.
35. Chang ER, Veeneklaas RM, Buitenwerf R, Bakker JP, Bouma TJ. To move or not to move: determinants of seed retention in a tidal marsh. Functional Ecology. 2008; 22: 720–727.
36. Chambert S, James CS. Sorting of Seeds by Hydrochory. River Research and Applications. 2009; 25: 48–61.
37. Clarke PJ, Kerrigan RA, Westphal CJ. Dispersal potential and early growth in 14 tropical mangroves: do early life history traits correlate with patterns of adult distribution? Journal of Ecology. 2001; 89: 648–659.
38. Iosilevskii G, Weihs D. Hydrodynamics of sailing of the Portuguese man-of-war *Physalia physalis*. Journal of the Royal Society Interface. 2009; 6: 613–626. doi: 10.1098/rsif.2008.0457 PMID: 19091687
39. Francis L. Sailing downwind: aerodynamic performance of the *Velella* sail. Journal of Experimental Biology. 1991; 158: 117–132.
40. Trakhtenbrot A, Nathan R, Perry G, Richardson DM. The importance of long-distance dispersal in biodiversity conservation. Diversity and Distributions. 2005; 11: 173–181.
41. Higgins SI, Richardson DM. Predicting plant migration rates in a changing world: The role of long-distance dispersal. American Naturalist. 1999; 153: 464–475.
42. Darwin C. On the Origin of Species. Oxford: Oxford University Press; 1859.
43. Dahdouh-Guebas F, Verneirt M, Cannicci S, Kairo JG, Tack JF, Koedam N. An exploratory study on grapsid crab zonation in Kenyan mangroves. Wetlands Ecology and Management. 2002; 10: 179–187.
44. Randin CF, Paulsen J, Vitasse Y, Kollas C, Wohlgemuth T, Zimmermann NE. Do the elevational limits of deciduous tree species match their thermal latitudinal limits? Global Ecology and Biogeography. 2013; 22: 913–923.
